# Q&A: Friends (but sometimes foes) within: the complex evolutionary ecology of symbioses between host and microbes

**DOI:** 10.1186/s12915-017-0455-6

**Published:** 2017-12-27

**Authors:** Nicole Gerardo, Gregory Hurst

**Affiliations:** 1 Department of Biology, Emory University, 1510 Clifton RD, Atlanta, Georgia 30322 USA; 2 Institute of Integrative Biology, University of Liverpool, Crown Street, Liverpool, L69 7ZB UK

## Abstract

Over the past decade, there has been a pronounced shift in the study of host–microbe associations, with recognition that many of these associations are beneficial, and often critical, for a diverse array of hosts. There may also be pronounced benefits for the microbes, though this is less well empirically understood. Significant progress has been made in understanding how ecology and evolution shape simple associations between hosts and one or a few microbial species, and this work can serve as a foundation to study the ecology and evolution of host associations with their often complex microbial communities (microbiomes).

## What is a symbiosis, a microbiome and a holobiont?

Broadly defined, symbioses are associations where one organism (the symbiont) lives with another (the host) over a considerable fraction of the life of the host. For host–microbe interactions, microbial symbionts reproduce many times within a host generation and persist over a significant fraction of the host's lifetime. By this definition, most microbes associated with infectious disease are not symbionts; they rapidly replicate and transmit to new hosts and then are cleared. Some hosts are associated with one or a few key symbionts. The ecology of others is shaped by association with a symbiotic community. These communities, and the genes that they contain, are referred to as microbiomes.

The term symbiosis dates to Anton de Bary [1]. More recently Margulis and Fester [2] coined the term ‘holobiont’, to reflect the individual organism as the sum of host and symbionts. Following a time when the term was rarely used, there has been a recent upsurge in popularity of the term and its use as a conceptual framework [3–5], driven by the desire to reflect the importance of microbes in animal life. This term has a genetic parallel, the hologenome—which represents the composite genetic information in host and symbionts [6, 7]. The utility of these terms has engendered wide debate and contrasting views amongst evolutionary biologists, ecologists, symbiosis researchers, and microbiologists [8, 9].

## When I think of microbiomes, I think of complex gut communities and fecal transplants. Is there more to it than that?

While the human microbiome project publically highlighted the complex bacterial community within the human gut, which we know plays an important role in digestion [10] and in disease susceptibility [11], the project also highlighted the complex microbial communities in other human body sites [12–14]. These too shape health and disease (for example [15]).

Diverse microbial consortia are seen in other host species as well, but are not a universal rule. The ecology and evolution of many host organisms are profoundly shaped by associations with one or a few dominant microbes. For example, almost all aphids, small, plant-feeding insects, associate with the bacterium *Buchnera aphidicola*, which critically synthesizes amino acids that aphids do not obtain from their plant sap diet [16, 17]. These bacteria are found not in the gut but within cells specifically adapted to house large numbers of this particular bacterium. Similarly, many legume plant species have nodules on their roots to house and maintain one or a few species of nitrogen-fixing bacteria. These bacteria profoundly shape their plants' fitness, particularly in nitrogen poor soils [18, 19].

## Why are people so excited about host associations with microbes?

Critical to the study of organismal biology is phenotype. We are interested in measuring traits of organisms, and then understanding the proximate (mechanistic) and ultimate (evolutionary) causes of those phenotypes. We study phenotype to explore how organisms interact with the world around them and to better understand disease (and how to treat it). We study phenotypic variation because population-level variation in heritable phenotypes is the foundation of evolution.

Research into an array of systems indicates that many organismal phenotypes are not simply the result of their genomes but are also influenced by their associations with microbes. Interactions with microbes fundamentally shape all life on earth, and natural phenotypic variation of multicellular organisms (and indeed many single-celled ones) is commonly associated with the presence of particular microbes or the structure of a host's associated microbial community. Experimentally, phenotype is commonly perturbed when organisms are reared axenically (for example [20, 21]), and in some cases, ridding an organism of its microbes leads to failure to develop or to produce viable offspring [22]. Understanding the phenotypic impacts of symbiosis on ecosystem function, food security and human health provides opportunities for improving well-being.

Variation in host phenotypes in nature can be driven by microbes in many ways. It may be underlain by the presence or absence of a certain microbe, the genotype of that microbe and, in more complex microbial communities, the variety and relative abundance of different community members [23]. In addition, absolute abundance of microbes may be important. The impact of abundance (titre) on phenotype has been recognised in studies of binary interactions between insects and heritable symbionts for some time (for example [24, 25]), and is now increasingly thought to be important in polymicrobial communities [26, 27]. The proximate causes and dynamics of all these levels of variation require explanation. Understanding their drivers may also facilitate manipulation of the community for therapeutic purposes (for example [11, 28]).

## Symbionts are good guys, right?

Broadly defined, symbionts are microbes that form long-term associations with their hosts—the microbes persist over a significant fraction of the host's lifetime and reproduce many times within a host generation. The long-term association contrasts with many pathogens, which arrive, replicate and are cleared (or kill their host). However, this does not mean that pathogens are bad and symbionts are good. Particular bacteria may be both persistent and deleterious, as is the case for *Clostridium difficile* infection [29]. Similarly, *Wolbachia pipientis*, bacteria found in a wide range of insect species, are transmitted only by females, and commonly shows sex-specific deleterious phenotypes, for instance killing male embryos produced by infected mothers. In this latter case, there is not a strong signature of pathogen-like disease systems in females, but carrying the microbe can nevertheless be costly [30].

Whether particular microbes (or microbial consortia) are beneficial or costly is often dependent on the ecological context of the association. For example, many animal and plant symbionts, from fungi growing within plant leaves [31, 32] to bacteria within insects [33], protect their hosts from infectious diseases. In the presence of the right disease agent, therefore, association with the protective microbe can have substantial fitness benefits. However, in the absence of the pathogen, the resource requirements of the symbiont may pose substantial costs to the host, such that the symbiont may decline in frequency in the population [34]. Indeed, where benefits are context-dependent, a host may try to reduce symbiont partner numbers to minimize costs. In legumes, for example, root nodules with bacteria that do not fix nitrogen are supplied with reduced resources, leading to reduction in symbiont titre [35].

It is often challenging to determine whether a symbiont provides a benefit or has associated costs. The fitness benefits to hosts are often explored by comparing the survival and reproduction of hosts when they do and do not have association with a particular microbe of interest. This can be simple to execute in systems where a single microbe can be cleared without perturbation to the rest of the microbial consortia (for example [36]). However, it remains challenging in many systems, where experimental clearance of one microbe is unavoidably coupled with disturbance of the larger microbial community. In addition, antibiotic use in clearance of microbes may have non-target effects that could give the appearance of a benefit to possessing the microbe. Tetracycline, for instance, impacts eukaryotic mitochondrial function, such that treated individuals both lose microbes and suffer direct physiological costs [37]. Furthermore, adaptation by a host to the presence of a microbe may lead to fitness being reduced on removal of the microbe, regardless of whether that microbe ancestrally contributed to host function [38].

## So what determines an individual’s microbial associates?

The microbial symbionts that are associated with an individual are a product of three processes: initial acquisition, replication within the host and loss through mortality or transmission to the environment. The mechanisms underlying these processes vary dramatically across species. In some hosts, like humans, microbes are acquired during birth and throughout life. Thus, the environmental microbial pool with which you make contact is one of the drivers of the microbes that you carry. Proximity of relatives makes transmission of symbionts between family members a relatively common event, and this may be particularly enhanced in the case of mother–offspring contacts [39]. However, that does not mean microbiomes are random assemblages derived from this contact network. Host genetic variation, immune responses, dietary preferences and the microbial community already within a host shape what is maintained and what is cleared [40, 41]. Community composition may also change through life—for example, as it does after weaning in mammals [42, 43].

The presence of microbial and host factors that structure assembly of microbiomes is evidenced in the phenomenon of *phylosymbiosis*—the tendency of related host species to have more similar microbial assemblages [44]. This pattern has been observed in multiple animal groups, from *Hydra* to mammals [45, 46]. Additionally, microbial community composition variation has a heritability within species, although commonly this explains only a minority of between-individual variation in composition [47].

In other systems, relationships are further tuned by specific mechanisms of microbe transmission from mother to offspring. Across insects, for example, microbes commonly pass from a female to her progeny. This mechanism of vertical transmission can be shaped by host traits to ensure transmission of beneficial symbionts to eggs or embryos (for example [48]). Alternatively, mothers may smear symbionts on egg surfaces or deposit them near eggs so that hatchlings will consume the microbes early in life [49, 50]. Indeed, some species exhibit specialized behaviours to search out these symbionts as soon as they are born [51]. Reciprocally, some microbes have evolved the capacity to infect host eggs and transmit vertically. *Wolbachia* in *Drosophila*, for instance, has the ability to invade the stem cell niches that generate the germline and soma of insect eggs [52].

## Why do parties evolve to be in symbiosis in the first place?

We can envisage several non-mutually exclusive evolutionary pathways towards symbiosis. The microbe may evolve from being free-living to being host-associated, or may evolve from being a pathogen to being a symbiont. Additionally, the host may evolve to nurture a particular microbe or microbes to its own benefit.

There is selection to enhance symbiosis when the relative fitness of the party in symbiosis is higher than that achieved outside symbiosis. For instance, if a microbe has higher fitness in symbiosis than in the environment, then selection will generally act to promote the symbiotic phase. Selection to enter into symbiosis may escalate where there are trade-offs between fitness in symbiosis compared to fitness out of symbiosis, such that adaptation to symbiosis reduces fitness of a microbe when free-living. There is good evidence for the presence of these trade-offs: *Vibrio fischeri* strains that perform well when in symbiosis with bobtail squid have lower environmental fitness than strains that are more poorly adapted to symbiosis but better adapted to the environmental phase [53]. The equivalent form for a host is higher fitness in symbiosis selecting for symbiont acquisition and maintenance, followed by adaptation to symbiont presence increasing the relative fitness difference between hosts with and without symbionts.

The processes of symbiont and host adaptation may be linked—evolution of a host to exploit a symbiont may be followed by evolution of the symbiont to be host-associated as it loses its environmental stage, and evolution of a microbe to be symbiotic may be followed by evolution of the host to maintain the symbiosis [54].

## What conditions favour the evolution of mutualism?

Mutualism is defined as an interaction in which both partners benefit from the association. Many symbioses that have known benefits for the hosts (at least under some ecological contexts) are presumed to be mutualisms. However, symbioses can be exploitative—one party derives benefit from the other without the other party benefitting in return [55]. In fact, we commonly have little quantitative understanding of the benefits, or costs, of symbioses for the microbes [56].

It is presumed that many symbiotic microbes derive fitness benefits through vertical transmission from one generation of hosts to the next, assuring representation in future generations. Furthermore, it is presumed that there is reduced competition in the host relative to the microbial warfare that encompasses most external environments. However, one cannot say whether the ancestor of the now host-associated symbiont had even higher reproduction, because we rarely know the details about the fitness of microbes or hosts at the initial stage of the evolution of these associations [56, 57].

What we do know is that there are a variety of circumstances in which the parties are more likely to evolve towards mutualism. The condition most propitious for driving symbioses towards mutualism is when the symbiont is vertically transmitted, passing from parent to offspring. Here, symbiont fitness is most tightly linked to the survival and reproduction of its host individual, leading to selection to improve host fitness [58]. As selection on the symbiont acts to improve host fitness, so the host is further selected to nurture the symbiont. However, even in these associations, fitness interests may not be completely aligned, and microbes and hosts may exploit each other (just enough) to maximize their own fitness [30, 59].

Nevertheless, mutualism can evolve without vertical transmission so long as the parties commonly interact. Partner choice, where one party recognises the other to form an interaction, further enables evolution to mutualism, enabling coevolution [60, 61]. For instance, the squid–*Vibrio* symbiosis is one in which host and microbe interaction is reformed each generation through specific colonization processes, and where squid and *Vibrio* fitness interests coincide, at least presumably with respect to host survival [62].

## Sometimes things evolve to be co-dependent—symbiotic fusions. Why is this?

As is clear, the lives of microbes and their host can become highly intertwined. The adaptation of one party to the other may result in dependence—an obligate requirement for the other party. For instance, a host may evolve its development or ecology within the context of symbiosis. Loss of the symbiont then leads to failure to develop or thrive. One of the earliest examples of a developmental requirement was recorded in *Psychotria* plants, which failed to develop when seeds were washed to remove bacteria (work of Miehe and von Faber, cited in [63]). It is now known that absence of symbiotic *Burkholderia* bacteria, which in adult plants form leaf nodules and are transmitted onto seeds, is the cause of this developmental failure [64, 65]. Remarkably, dependence can evolve rapidly, as has been observed during laboratory studies of amoeba–microbe interactions [66].

The principles applied to hosts apply equally to symbionts. Genome reduction (compared to the free living ancestor) occurs rapidly following the evolution of intracellular symbiosis [67]. Gene loss may be associated with selection for loss of pathways provided by partners [68, 69], or accumulation of deleterious mutations associated with restricted population size and absence of opportunity for recombination [70]. Loss of pathways creates an evolutionary trajectory in which the symbionts become obligately host-associated.

## Does dependence make things more resilient, or are there negative consequences?

Dependent relationships can be long-lived—as is the case of mitochondria in eukaryotes and chloroplasts in plants. However, whether symbiotic fusions necessarily create more persistent lineages is unclear. Dependence of a host upon a symbiont may reduce resilience, as the host is affected not just by the factors that affect it but also by those affecting its symbiont. Furthermore, dependent symbionts often become a weak link because of genome degradation. *Buchnera*, the bacterium upon which aphids are dependent, for example, has many poorly functioning proteins as a result of mutation accumulation [70, 71]. High temperatures destabilize *Buchnera* proteins, making the required symbiont the ‘weak link’ under thermal stress [72].

Things get more complicated when more than one symbiont is involved. Models of polymicrobial communities indicate strong mutualistic/dependent interactions between microbes are destabilizing to microbiome composition. This is because loss of one of a pair of mutualistic microbes makes the other prone to loss. Indeed, it is predicted microbiome stability is higher under competition between microbe strains rather than under mutualism [73].

## What are the most pressing questions in the study of the evolutionary ecology of host–symbiont interactions?

Our understanding of the evolutionary ecology of binary interactions (that is, between a host and a particular symbiont with which it is commonly associated) is well-developed compared to our understanding of the assembly and evolution of complex polymicrobial communities. It is recognised that a ‘bottom up’ approach, where models build from functionality to predict community dynamics, will be particularly useful for predicting microbiome dynamics and evolution [74, 75].However, the complexity inherent in these models, and lack of well-founded values for many of the parameters, have to date limited the utility of this approach. Instead, there has been the development of a more pragmatic framework that has predicted community dynamics from observed statistical associations: which taxa have abundances that are positively/negatively associated; how abundance varies with diet [76, 77]. A shortcoming of these models is that they have a weaker conceptual basis, and as a result may predict the response to uncharacterized events poorly.

In the absence of a strong predictive theoretical basis, can we address microbiome impacts and dynamics empirically instead? In terms of phenotype, there is increasing functional knowledge of particular microbes—the resources that they utilize and the metabolites that they secrete [78]. Our understanding of host genetic variants associated with microbiome variation is also increasing [79]. However, causal flows linking the microbiome to host phenotype are sometimes elusive. Much is still based on association of microbiome constitution with phenotype, and, as for all statistical associations, these have three potential causal flows: microbiome drives phenotype, phenotype drives microbiome or an external agent drives both. Experimental tests of causation are difficult; however, manipulation experiments, in which the impact of microbial community transplants are measured, have allowed causal inference [80].

Whilst progress is being made to define the impact of microbiomes on phenotypes, many core parameters remain poorly described. For example, evolutionary theory tells us the tendency towards beneficial interactions and partnership increases with prolonged partner association, and also depends on transmission pathways. Both of these parameters are commonly poorly understood. With respect to the length of association, we generally do not know how long a particular symbiont lineage (rather than symbionts within a species cluster) is retained in a host individual. As a corollary, we know turnover occurs within microbiomes, but less about the relative importance of the factors that may drive this change. Is it external change such as diet or pathogens, or is it a form of succession and turnover driven internally? The second key parameter—transmission—is also commonly poorly understood. Whether transmission occurs to progeny, relatives, members of the same species or heterospecifics is of key importance in determining evolutionary dynamics. Further, whether transmission occurs directly or following replication in the environment is important in determining the importance of the symbiotic state to microbe fitness. Despite the importance of the transmission process in determining evolutionary trajectories, we do not have a clear understanding of transmission in most systems with complex communities [81]. Uncovering the assembly and dynamics of microbiomes within individuals remains a major evolutionary challenge, and will be critical as we move to understand their importance in shaping host phenotype and host evolution.
